# Linguistic and Clinical Validation of the Tajik Acute Cystitis Symptom Score for Diagnosis and Patient-Reported Outcome in Acute Uncomplicated Cystitis

**DOI:** 10.3390/medicina59091549

**Published:** 2023-08-25

**Authors:** Abdukhamid Radzhabov, Musluhuddin Zamuddinov, Jakhongir F. Alidjanov, Adrian Pilatz, Florian M. Wagenlehner, Kurt G. Naber

**Affiliations:** 1 Department of Urology, Municipal Hospital, 38118 Brunswick, Germany; abduradzhabov@gmail.com; 2 Departments of Urology, Madadi Akbar Clinic, Dushanbe 734025, Tajikistan; zamuddinov86@mail.ru; 3 Departments of Urology, Olami Tib Clinic, Dushanbe 734060, Tajikistan; 4 Clinic for Urology, Pediatric Urology and Andrology, Justus-Liebig University, 35392 Giessen, Germany; jakhongir.alidjanov@med.uni-giessen.de (J.F.A.); adrian.pilatz@chiru.med.uni-giessen.de (A.P.); florian.wagenlehner@chiru.med.uni-giessen.de (F.M.W.); 5 Department of Urology, Technical University of Munich, 80333 Munich, Germany

**Keywords:** urinary tract infection, cystitis, Acute Cystitis Symptom Score, ACSS, patient-reported outcome, typical symptoms, quality of life

## Abstract

*Background and Objectives*: Acute Cystitis Symptom Score (ACSS) is a self-reporting questionnaire for clinical diagnosis and follow-up of acute uncomplicated cystitis (AC) in women. The ACSS, originally developed in Uzbek and Russian, both considered original languages, is now available in several other languages. This study aimed to translate and validate the ACSS in the Tajik language. *Material and Methods*: Linguistic validation was carried out according to the Linguistic Validation Manual for Patient-Reported Outcomes Instruments guidelines. Clinical validation was performed by enrolling fifty-four Tajik-speaking women. All women included in this study were first interviewed about the understandability of all questions and statements in the final Tajik ACSS and were asked to fill in form A at the first visit (diagnostics) and form B at any follow-up visit (patient-reported outcome). *Results*: Thirty-three women, median (range) age of 35 (18–77), were diagnosed with AC (patient group), while twenty-one women, median (range) age of 34 (20–61) (*p* = 0.109), were enrolled as the control group without any other urological disease. For the diagnostics of AC, a summary score of the six typical symptoms (“Typical” domain) showed the best balance between sensitivity (0.73) and specificity (0.71) at 5 and above. Cronbach’s alpha [95% CI] and split-half reliability [95%] were 0.82 [0.76; 0.98] and 0.84 [0.77; 0.87], respectively. At the follow-up visit, the patients reported a significant reduction in the “Typical” domain and an improvement in the “Quality of Life” domain. *Conclusion*: The Tajik ACSS showed good reliability and diagnostic values and may be used as a reliable tool for the diagnosis and patient-reported outcome in women with AC in clinical and epidemiological studies and for daily practice.

## 1. Introduction

Acute uncomplicated cystitis is one of the most common infectious diseases within the female population. About 20–30% of adult women have an episode of dysuric symptoms once or more times a year, half of which corresponds to a urinary bladder infection [1]. The local and generalized symptoms of acute uncomplicated cystitis (AC) impact their quality of life (QoL), daily activity, and psycho-emotional state [1,2,3,4].

Wagenlehner et al. [5] conducted a multinational study in Europe (Germany, Switzerland, Poland, Russia, and Italy; GESPRIT) to analyze the social and economic burden and the impact of recurrent urinary tract infection (UTI) on QoL, defined as two or more acute episodes within six months or three or more acute episodes within one year. Using the SF-12v2 questionnaires, the data from their study indicated that recurrent UTI in all five countries has a detrimental effect on QoL and is associated with mental stress for a high proportion of affected women, which also might cause depression. These findings support the data by Renard et al. [6], who found that around 62% of patients with recurrent UTI reported some degree of depression and that effective prophylaxis significantly improved their QoL [6].

Various urinary symptoms have been used to assess the diagnosis and severity of acute uncomplicated cystitis in women [7,8,9,10,11], but only a few studies developed questionnaires to also evaluate the severity and impact on activity impairment [9,10], which, however, were not designed for diagnostics of AC, but only for follow up.

According to the European Association of Urology Guidelines 2022, uncomplicated cystitis is defined as acute, sporadic, or recurrent cystitis limited to non-pregnant women with no known relevant anatomical and functional abnormalities within the urinary tract or (relevant) comorbidities. The diagnosis can be based on a focused history of lower urinary tract symptoms (LUTS), such as dysuria, frequency, and urgency, in the absence of vaginal discharge. Urine tests or other microbiological investigations are not always considered necessary for the first episode of acute uncomplicated cystitis [12]. According to the guidelines by the European Medicines Agency (EMA) and US Food and Drug Administration (FDA), female patients with AC should have a minimum number of symptoms such as frequency, urgency, and dysuria, and according to FDA guidelines, at least two of the following four symptoms: frequency, urgency, dysuria, and suprapubic pain. Patients may be enrolled in studies on the evaluation of medicinal products before microbiological culture results are available based on documented pyuria (≥10 white blood cells (WBCs) per µL) in a mid-stream specimen [13,14]. Nevertheless, the perception of symptoms is paramount for the diagnosis of an acute episode of uncomplicated cystitis. However, most patients pre-diagnose themselves with bladder inflammation based on general symptoms such as increased frequency, urgency, and dysuria, but often they associate it with colds.

The Acute Cystitis Symptom Score (ACSS) was developed to simplify the diagnosis and timely treatment of acute uncomplicated cystitis in women, facilitating physicians’ practice of treating these patients. The ACSS was first developed in Uzbek and later translated into other languages, including Russian, German, UK and US English, French, Greek, Hungarian, Italian, and Ukrainian (http://www.acss.world, accessed on 20 August 2023), and was used in different clinical studies [15,16,17,18,19,20,21,22,23]. Since the initial Uzbek version and the linguistically validated Russian version were clinically tested in Uzbekistan at the same time, because both languages were used in the country with about the same frequency, the Russian version was also considered the original version of the ACSS [15].

The ACSS consists of two parts (Table 1, Supplementary Tables S1–S3) [15,20]: part A is used at the first visit for diagnosis and part B at any follow-up visit. Part A includes 18 questions grouped into four sections: (1) the typical symptoms of acute cystitis (frequency, urgency, dysuria, incomplete bladder emptying, suprapubic pain, and visible blood in urine), (2) differential diagnosis (flank pain, vaginal and urethral discharge, and high body temperature/fever), (3) patient quality of life (discomfort because of the symptoms, interference with everyday activities or work, and interference with social activities), and (4) additional circumstances (menstruation, premenstrual syndrome, menopausal syndrome, pregnancy, and known diabetes mellitus) that may affect therapy tactics. Each of the questions in the first three sections (typical symptoms, differential symptoms, and quality of life) can be answered with none, mild, moderate, or severe. Part B, used at any follow-up visit, inquires in the first section about the general changes in the symptoms compared to the first visit (overall patient assessment), followed by the four sections of part A. As the patient is always asked about the severity of each of the typical or differential symptoms and her quality of life (parts A and B), she will develop a personal score of severity, which is, of course, subjective, but on a personal scale.

The aim of this prospective study was not only to translate the ACSS from the original Russian into the target Tajik language following accepted international guidelines but also to cognitively assess the Tajik version of the ACSS by interviewing study participants and to validate it clinically under real-life conditions.

## 2. Results

After translating according to the accepted international guidelines [24,25,26] from the original source language, Russian [15] (Supplementary Table S2), into Tajik as the target language, the final Tajik version of the ACSS was used for the clinical study (Table 1, Supplementary Table S3).

The recruitment of participants for this clinical validation study resulted in a total of fifty-eight women seeking medical care at two urology departments, one at the Madadi Akbar Clinic and the other at the Olami Tib Clinic, both in Dushanbe, Tajikistan. Fifty-four were considered suitable for statistical analysis, and four were excluded because of missing data. Thirty-three respondents (61.1%) with a diagnosis of AC by the attending physician at the time of admission represented the patient group, and twenty-one (38.9%) without urological disorders represented the control group. A total of 54 questionnaires were completed at baseline and follow up. The median (IQR) time between visits was 11.0 (10.0–14.0) days (range 6–41 days), with 11.0 (10.0–11.2) days for the control group and 12.0 (9.0–15.0) days for the patient group (*p* = 0.379).

The median (IQR) age of the patient group was 35.0 (25.0–58.0) years (range 18–77 years) and 34.0 (27.0–41.0) years (range 20–61 years) for the control group (Table 2). Significant differences (*p* < 0.05) were generally found between the patient and control groups in the presence of the five typical symptoms of AC and the negative impact on QoL. Cronbach’s alpha and split-half reliability were high for the “Typical” and “QoL” domains but, as expected, not for “Differential” (Table 3).

The diagnostic values for a summary score of typical symptoms (“Typical” domain) showed the best balance between sensitivity (0.73) and specificity (0.71) at 5 and above, whereas at 6 and above, specificity (0.76) was higher and sensitivity (0.67) lower. However, the positive predictive values were similar at both cut-offs (0.80 vs. 0.81) (Table 4).

**Figure 1:**
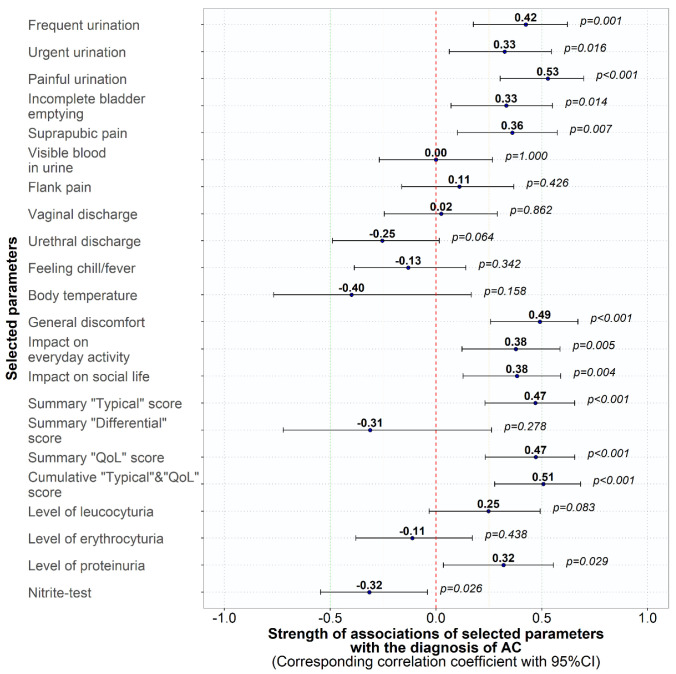
Correlation coefficient (95% CI) between ACSS and urinalysis items and the diagnosis of acute uncomplicated cystitis according to the treating physician.

Of the typical symptoms, painful urination showed the highest correlation with the diagnosis of AC, although this correlation was also significant for the other typical symptoms but not for “visible blood in urine”, which was only observed in 9 (27.3%) of the patients with AC and 4 (19.0%) of the negative controls without urological disease. The reduction in QoL was also significantly correlated with the diagnosis of AC in the patient group in all three categories (Figure 1 and Table 2). Of the urinalysis parameters, neither leukocyturia, erythrocyturia, nor a positive nitrite test correlated significantly with the diagnosis of AC.

**Figure 2:**
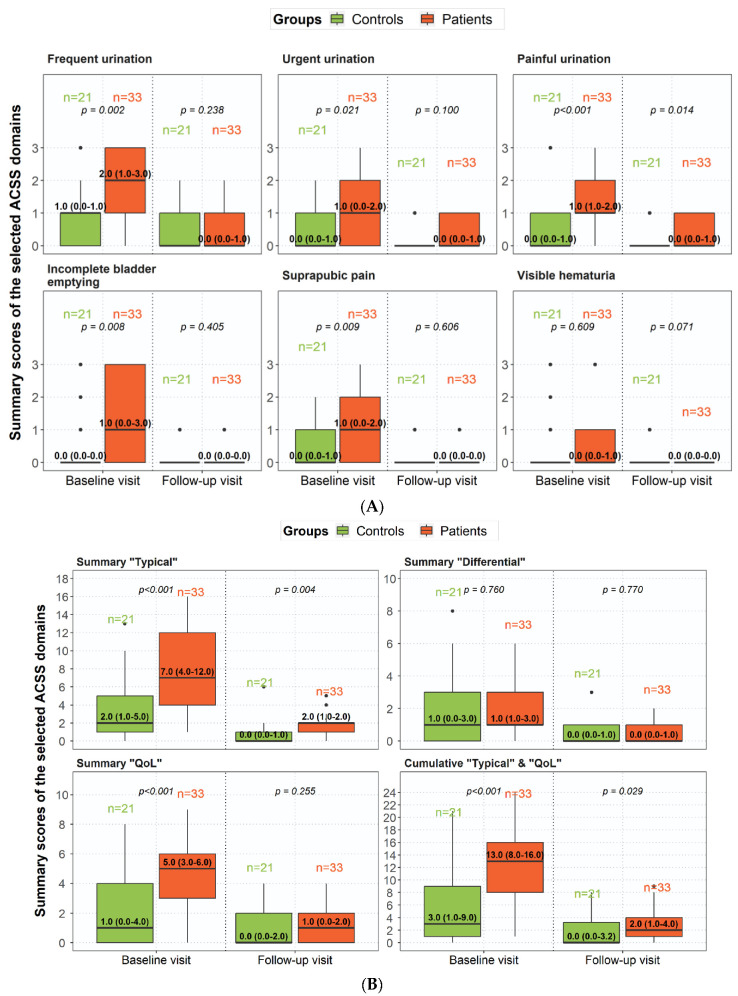
(**A**) Severity (scores) of ACSS “Typical” symptoms comparing controls and patients according to the diagnosis of the treating physician (median ± quartile, range). *p* < 0.05 significant. (**B**) Severity (summary scores) of ACSS “Domains” symptoms comparing controls and patients according to the diagnosis of the treating physician (median + quartile, range). *p* < 0.05 significant.

Figure 2A,B present the severity (scores) of individual symptoms and ACSS domains at baseline and follow-up visits in the patient group and at baseline in the control group. Again, the patient group had significantly higher scores than the control group at baseline, except for visible blood in urine and the “Differential” domain. At the follow-up visit, all parameters showed a significant reduction in the patient group, while, in general, the severity rates in the patient group no longer differed from those in the control group. Only for painful urination was the severity slightly but significantly higher in the patient group than in the control group at the follow-up visit and, consequently, in the “Typical” domain.

**Figure 3:**
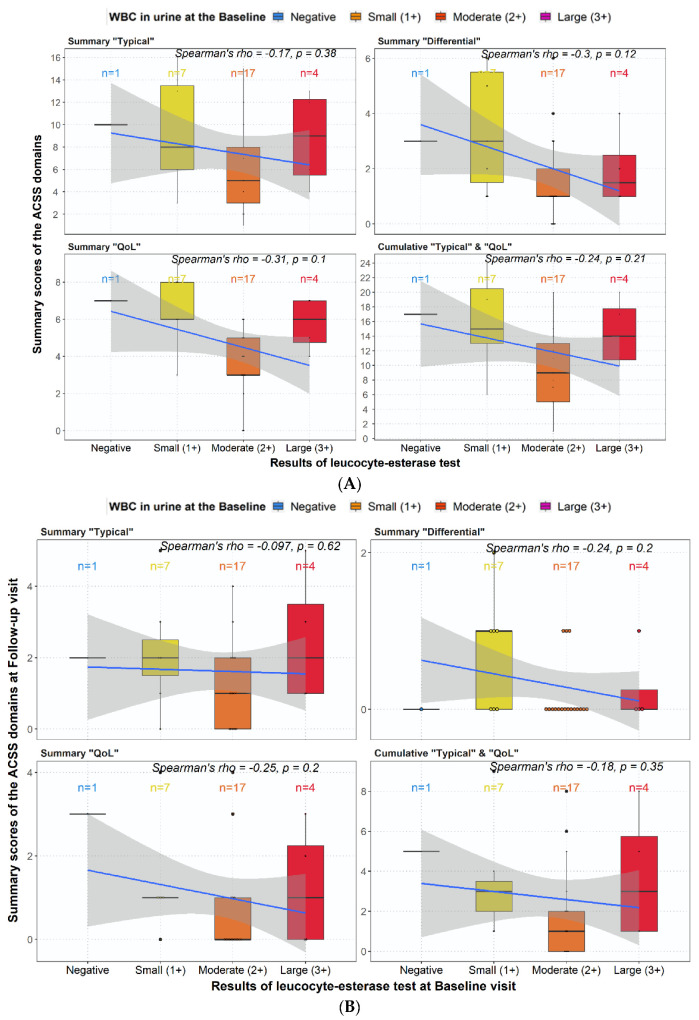

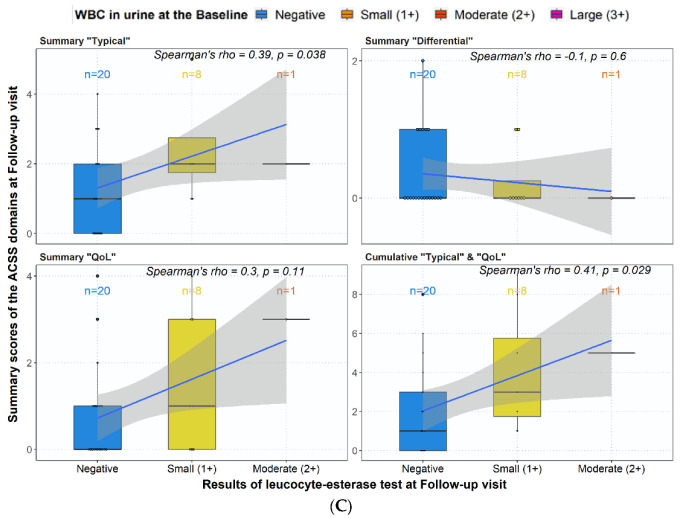
Correlation between (**A**) summary scores of ACSS domains and amount of leukocyturia at baseline visit; (**B**) summary score of ACSS domains at follow-up visit and amount of leukocyturia at baseline visit; (**C**) summary scores of ACSS domains and amount of leukocyturia at follow-up visit. Of note: in 3 patients, urine was not checked for leukocyturia. Note: The color fields correspond to the following values; Negative/Trace = <10 WBC/µL, Small (1+) = approx 25 WBC/µL, Moderate (2+) = approx. 75 WBC/µL, and Large (3+) = approx. ≥500 leukocytes/µL. The blue line represents the trend line of logistic regression, and the grey area around represents the standard error (SE).

The amount of leukocyturia at baseline had no significant positive correlation with the severity (scores) of the ACSS “Domains” at the baseline visit (Figure 3A) and no predictive value for the outcome at the follow-up visit (Figure 3B). The amount of leukocyturia at the follow-up visit, however, correlated with the severity (scores) of the “Typical” domain (Figure 3C) but not with the “Differential” and “QoL” domains.

**Figure 4:**
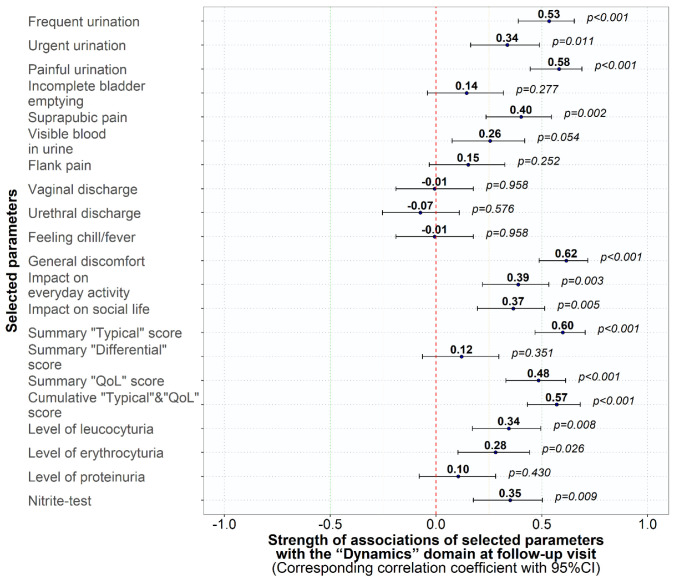
Association coefficient (95% CI) between ACSS and urinalysis items and the ACSS “Dynamics” domain.

**Figure 5:**
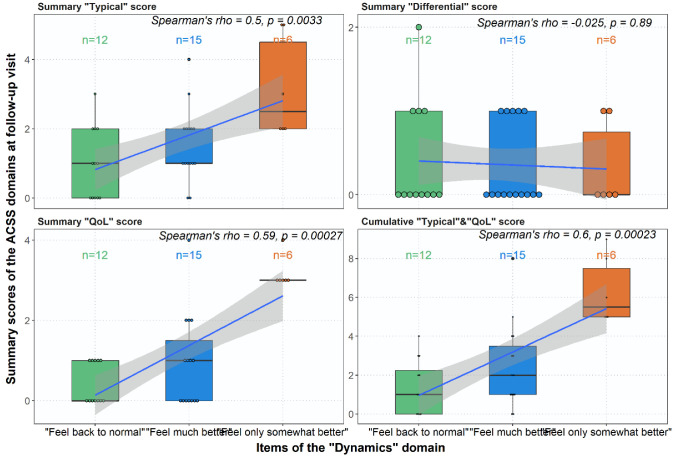
Correlation between scores of ACSS domains and “Dynamics” in patients at follow-up visit. Three “Dynamics” categories were reported at follow-up visit: 0—feel back to normal; 1—feel much better; 2—feel only somewhat better.

At the follow-up visit, the ACSS is also used as a patient-reported outcome measure (PROM) using different categories. In the “Dynamic” domain, the patient reports her overall situation in case of successful treatment, whether she feels back to normal because all symptoms were gone or she feels much better. In contrast, feeling only somewhat better, feeling no change, or even worse can only be considered a clinical failure. When the overall clinical assessment by the patient (“Dynamics” domain) was correlated with the severity (scores) of the other ACSS items, a significant correlation could be shown with the severity of all five typical symptoms, the three categories of the “QoL” domain, but also with the amount of leukocyturia, erythrocyturia and a positive nitrite-test (Figure 4). The significant correlations between the three “Dynamics” categories found in the present study with the “Typical” and “QoL” domains, but with the “Differential” domain, is also shown in Figure 5.

## 3. Discussion

### 3.1. About the Tajik Language

Tajik is the official language of Tajikistan and is considered the native language of about 80% of the country’s 9.5 million population. The Tajik language is a West Iranian language and is spoken in Tajikistan, Uzbekistan, and Kyrgyzstan [27,28,29].

In the 8th century AD, the Persian-speaking natives of Khorasan, who were converted to Islam, actively participated in the Arab conquest of Central Asia. As a result, the Persian language had gradually, for 2–3 centuries, replaced local languages (Sogdian, Bactrian, and others) in most of Central Asia. Subsequently, the Persian language in Central Asia had also been influenced by neighboring Turk languages. The Persian-speaking population of Central Asia was called the “Tajik”, although the language of the Central Asian Tajiks continued to be called “Farsi” (Persian) until the twenties of the 20th century when Tajikistan was given the status of a republic and the Persian language was renamed to the Tajik language during the Soviet time mainly for political and ideological reasons in order not be associated with “Persian King”.

Ethnic Tajiks make up the majority of the population in Bukhara and Samarqand, two of the oldest and biggest cities of Uzbekistan. Many ethnic Tajiks also live in the Surkhondaryo Region of Uzbekistan in the south and the Uzbek part of Fergana Valley in the north.

The Tajik language is close to the Persian Dari language spoken in Afghanistan by Tajiks, who make up most of the population in northern Afghanistan and other parts of the country, particularly in large cities. A relatively small diaspora of Tajiks lives in Kyrgyzstan. In addition, a sizeable Tajik-speaking diaspora exists in the Russian Federation, where about one million Tajiks work as migrant laborers because of the poor socio-economic situation and the high unemployment rate in Tajikistan.

The numerous dialects of the Tajik language can be divided into Northern (Sughd province, Bukhoro and Samarkand cities of Uzbekistan, and Kadamjay district of Kyrgyzstan), Central (Zarafshan and Varzob Valleys), Southern (Hisor, Khatlon, and Rasht), and Southeastern (Darvoz and Vanj) dialects. In addition, most of the small population of Gorno-Badakhshan Autonomous Province (GBAO) speaks diverse Pamiri dialects of the Tajik language spoken by about 250 thousand people.

Today’s Tajik script uses the Cyrillic alphabet since 1939; before that, until 1928, the Arabic alphabet, and from 1928 to 1939, the Latin alphabet.

### 3.2. Linguistic and Clinical Validation

The linguistic and clinical validation of the Tajik ACSS was conducted according to internationally accepted guidelines, as for the previous languages [24,25,26]. In contrast to other languages, the Tajik ACSS showed the best diagnostic balance between sensitivity and specificity at a summary score of typical symptoms of 5 rather than 6 and above. This small difference may not be significant, but more important, the sensitivity at this summary score was only 0.72 and the specificity 0.73, rather than over 0.80 or even around 0.90 as in other languages (Table 5).

Although this clinical validation study only included patients who had not taken antibiotics in the previous 3 months for any reason, we could not find any comment in the study material about the previous use of symptomatic treatment in the patient’s case history. In Tajikistan, however, patients with complaints of UTI symptoms do not usually seek medical care during the early stages. This may be due to living in remote areas or the lack of specialized medical facilities nearby where diagnosis and appropriate laboratory tests could be carried out. As a result, most patients start “self-indicated” herbal or other treatments without consulting a physician.

In this context, it is of interest that all patients included in this study had experienced at least one symptomatic episode of AC in the last year, although only nine had recurrent UTI according to the following definition: at least two episodes in the last 6 months or three episodes in the last year. However, all patients have already experienced the symptoms of AC at some point in their lives. Thus, it is reasonable to assume that at least some of these patients have started symptomatic therapy before their first visit to the treating physician. This may explain why some patients had already experienced a reduction in symptom severity, as shown in a reduced summary score of the “Typical” domain. As this current study was designed as a prospective observational study, urine culture was not performed to confirm the diagnosis at the baseline visit or patient-reported outcome at the follow-up visit.

The observation may be of Interest that the amount of leukocyturia does not correlate with the severity of symptoms, and the diagnosis of AC also does not predict outcomes, as shown in another study as well [23]. However, patients who felt only somewhat better at the follow-up visit had a higher amount of leukocyturia than those who felt much better or were back to normal because all symptoms were gone (ACSS domain “Dynamics”) (Figure 3C).

Finally, the clinical treatment outcome of the patients included in this study was similar to former studies if the same thresholds for successful treatment were used [22]. For clinical studies using non-antibiotic treatment, e.g., as a comparator, clinical improvement rather than the elimination of bacteriuria should become the main study aim. In such studies, the ACSS could be used as a well-defined patient-reported outcome measure (PROM).

### 3.3. Limitations

The limitations of our study can be summarized as it is a prospective, observational, i.e., non-interventional, real-world study carried out in only two urological clinics in Tajikistan, including a relatively small number of female patients with signs and symptoms of AC (n = 33) and those without any other urological diseases as a control group (n = 21). Whether the differences observed in the severity of symptoms in the group of patients diagnosed with AC as compared with studies in other countries/languages can only be explained as cultural or organizational differences in the health care system, as mentioned above, remains open. Another explanation for the relatively low sensitivity and specificity of the diagnostic value in this study compared to other countries (Table 5) may be explained via the inclusion of individuals in the patient group who had received some form of symptomatic therapy before attending the urology clinic. Therefore, further studies in Tajikistan are recommended to include a sufficient number of patients who have not been pre-treated for acute cystitis.

### 3.4. Lessons Learned from this Particular Study

Since, in general practice, patients with AC are usually diagnosed based on clinical symptoms, probably including only urinalysis but usually not urine culture, it is obvious that the correct assessment of the clinical symptoms becomes more important. As studies have shown that some kind of symptomatic therapy for AC may become an accepted alternative to antibiotic therapy [19], the scientific development of such questionnaires as the ACSS becomes even more important because of not only the presence but also the severity of the clinical symptoms is important for the diagnosis as well as for the PROM of AC [11,21,22].

## 4. Materials and Methods

### 4.1. Development of the Tajik Version of the ACSS

The translation of the Tajik version was carried out according to accepted international guidelines [24,25,26] with the following steps: the initial Russian version of the ACSS [15] was first translated into Tajik (the target language) by two professional translators with Tajik as their mother language. The translators were blinded to each other and worked independently. The translated versions were discussed by the research team (authors), and after making the necessary corrections, the consented version was translated back into Russian by an independent professional Russian-speaking translator. The Russian-speaking coauthor compared the backward translated version with the original Russian version of the ACSS.

After discussion with the translators and approval by the research team, consisting of the authors and two additional urological colleagues working in the two Tajik clinics, the final Tajik version has been recognized as final for this clinical study (Table 1).

### 4.2. Study Design, Study Procedures, and Clinical Investigation

This study was designed as a prospective, observational clinical trial conducted in Tajikistan and included Tajik-speaking women from November 2017 to February 2019. After the treating physician made the diagnosis of AC based on the clinical investigations, including routine physical examination, medical history, and urine dipstick test (Combur-Test^®^ 10) to estimate pH, the amount of white and red blood cells, and protein (WBC, RBC, PRO) in urine, the patients with acute uncomplicated cystitis (patient group) and women without urological diseases (control group) were recruited for this study. The control group included women who attended the clinic for gastroenterological, cardiovascular, and orthopedic complaints without any indication of hospitalization. For none of the women in the control group, the treating physician diagnosed any kind of UTI. The study participants in both groups were women of different levels of education, such as post-secondary, post-secondary non-tertiary, and tertiary education levels. Women who agreed to take part in this study were referred to the independent research team, consisting of three urologists working in the two Tajik clinics, to be informed in more detail about this study and to give their signed informed consent.

After the participants were enrolled in this study and had completed a demographic questionnaire, they were given the final Tajik version of the ACSS. They were asked if they had any cognitive difficulties understanding any of the questions or statements within the ACSS. Once the research team was assured that there were no problems with the cognitive assessment of the study respondents, the women of the patient and control groups were asked to fill in part A (diagnostic part) of the ACSS at the baseline visit and part B (patient-reported outcome) at each follow-up visit. All data collected were recorded in a secure online database using specially developed software.

### 4.3. Data Processing

The personal data of all participating women were pseudonymized. As reference variables to control whether the “real-life” diagnosis made by the treating physicians matches the “defined” diagnosis, the current definitions of uncomplicated UTI proposed by EMA and FDA (revision 3) were used [13,14]. The number of leukocytes (WBC) in the urine according to the dipstick test was dichotomously allocated as pyuria negative (dipstick test “Negative” or “Trace”: <10 WBC/µL) and pyuria positive (“Small” (1+): approx. 25 WBC/µL; “Moderate” (2+): approx. 75 WBC/µL; “Large” (3+): approx. 500 WBC/µL) [30].

### 4.4. Statistical Analysis

The sample size calculation for the validation of the 18 questions at the *p*-value of 0.049 and the power of 0.90 with confidence level of 95% based on the prevalence of the exposure of 0.33 resulted in a minimum population of 28.7 per arm [31,32]. Normality of distributions, linearity, and homoscedasticity of data was tested visually (using histograms, normal Q-Q Plots, etc.) and mathematically using Shapiro–Wilk and Levene’s tests [33,34].

Numerical values were presented via measures of central tendency (e.g., mean, median), distribution, and dispersion (e.g., standard deviation, 95% confidence intervals, interquartile range). A comparative analysis of the independent continuous variables was performed using a two-sided independent sample *t*-test and paired *t*-test with the Welch correction in cases of inequality of variances [35]. Finally, categorical variables were presented in proportions and compared using McNemar’s test [36].

The reliability of the ACSS and its domains was assessed via the internal consistency of the items and represented using Cronbach’s alpha and the split-half reliability coefficients [37]. The discriminative ability of the ACSS was assessed by comparing the respective item scores and summary scores of the ACSS domains between patients and controls at the baseline and follow-up visits.

Responsiveness of the ACSS domains was measured by comparing respondents’ summary scores on different domains of the ACSS at baseline and follow-up visits and the changes in scores of the “Dynamics” domain at various follow-up visits. Diagnostic values of the domains and items of the ACSS are presented using appropriate average values of sensitivity, specificity, positive and negative predictive values, diagnostic odds ratio (DOR), and Youden’s index.

Comparisons of ordinal and interval variables and values of matched groups (e.g., total scores of patients on ACSS domains at baseline and follow-up visits) were performed using the Wilcoxon signed-rank test [38]. The strength of associations was measured depending on the nature of the variables. In general, we used Pearson’s product-moment correlation coefficient (r) for continuous variables, Kendall’s and Spearman’s rank correlation coefficients for ordinal variables, and point-biserial and tetrachoric correlation coefficients for dichotomous ordinal values [39,40,41,42,43,44]. Statistical significance was set at 0.05.

R-Studio with integrated R and in-built and additional packages were used for the analysis and graphical representation of the results [44,45,46,47,48,49].

## 5. Conclusions

The ACSS, a standardized self-reporting diagnostic questionnaire for clinical diagnosis, differential diagnosis, and patient-reported outcome measure in women with AC, can be recommended for epidemiological and interventional studies but also allows for women to self-diagnose acute uncomplicated cystitis. Since the Tajik version of the ACSS was linguistically validated according to international guidelines and cognitively assessed by interviewing all-female study participants of different ages and educational levels, it was also clinically validated in a prospective, observational study under real-world conditions and can be used in clinical studies and everyday clinical practice.

## 6. Patents

The ACSS is copyrighted by the Certificate of Deposit of Intellectual Property in Fundamental Library of the Academy of Sciences of the Republic of Uzbekistan, Tashkent (Registration number 2463; 26 August 2015) and the Certificate of the International Online Copyright Office, European Depository, Berlin, Germany (Nr. EU-01-000764; 21 October 2015).

The rightsholders are Jakhongir Fatikhovich Alidjanov (Uzbekistan), Ozoda Takhirovna Alidjanova (Uzbekistan), Adrian Martin Erich Pilatz (Germany), Kurt Guenther Naber (Germany), and Florian Martin Erich Wagenlehner (Germany).

The e-USQOLAT is copyrighted by the Authorship Certificate of the International Online Copyright Office, European Depository, Berlin, Germany (Nr. EC-01-001179; 18 May 2017) 19.

Translations of the ACSS in other languages are available on the website: http://www.acss.world/downloads.html (accessed on 20 August 2023).

## Figures and Tables

**Table 1:** Tajik version of the Acute Cystitis Symptom Score (ACSS)—Савoлнoмаи ACSS.

First Visit—Part A (Diagnostic Part)			Time: :	Date of Evaluation: / / (mm/dd/yyyy)		
Ташрифи аввалин—Қисми А (“ташхисӣ”)			Вақт: :	Санаи пур кардани савoлнoма: / / (рӯз/мoҳ/сoл)		
Хoҳишмандем нишoн диҳед, ки oё Шумo алoматҳoи зеринрo дар давoми 24 сoати oхир ҳис намудед ва дараҷаи таъсири oнҳoрo баҳo диҳед *(Танҳo барoи ҳар як алoмат як ҷавoб диҳед)*:						
			0	1	2	3
**Алoматҳoи типӣ**	**1**	Тез-тез пешoбкунӣ бo ҳаҷми кам *(тез-тез ба ҳoҷатхoна рафтан)*	□ Не *тo 4 марoтиба* *дар 1 рӯз*	□ Бале, нисбат ба ҳарвақта бештар *5–6 марoтиба* *дар 1 рӯз*	□ Бале, зудтар пайдo мешавад *7–8 марoтиба* *дар 1 рӯз*	□ Бале, тез-тез пайдo мешавад *9–10 марoтиба дар 1 рӯз*
	**2**	Майли бетoқат пешoбкунӣ (*сахт ва нигoҳдoштанашаванда)*	□ Не	□ Ҳа, суст	□ Ҳа, муътадил	□ Ҳа, сахт
	**3**	Дард ё сӯзиш ҳангoми пешoбкунӣ	□ Не	□ Ҳа, суст	□ Ҳа, муътадил	□ Ҳа, сахт
	**4**	Ҳиссиёти пурра хoли нашудани пешoбдoн	□ Не	□ Ҳа, суст	□ Ҳа, муътадил	□ Ҳа, сахт
	**5**	Дард ва нoрoҳати дар қисми пoёни шикам *(дар қисми бoлoии зери нoф)*	□ Не	□ Ҳа, суст	□ Ҳа, муътадил	□ Ҳа, сахт
	**6**	Мавҷуд будани хун дар пешoб	□ Не	□ Ҳа, суст	□ Ҳа, муътадил	□ Ҳа, сахт
			**Миқдoри умумии хoлҳo “ Типӣ ”=**			**хoл**
**Дифференциалӣ**	**7**	Дард дар қисмати камарбанд * *—бисёртар аз як тараф	□ Не	□ Ҳа, суст	□ Ҳа, муътадил	□ Ҳа, сахт
	**8**	Пайдoшавии зардoб дар узвҳoи танoсул *(асoсан саҳаргoҳoн)*	□ Не	□ Ҳа, суст	□ Ҳа, муътадил	□ Ҳа, сахт
	**9**	Пайдoшавии зардoб аз рoҳҳoи пешoбрав *(нoвoбаста аз пешoбкунӣ)*	□ Не	□ Ҳа, суст	□ Ҳа, муътадил	□ Ҳа, сахт
	**10**	Ҳарoрати баланди бадан *(бештар аз 37.5 °С)*/ларза	□ Не ≤37.5 °C	□ Ҳа, суст 37.6–37.9 °C	□ Ҳа, муътадил 38.0–38.9 °C	□ Ҳа, сахт ≥39.0 °C
		*(Агар чен карда бoшед интихoб намoед)*				
			**Миқдoри умумии хoлҳo “Дифференциалӣ”=**			**хoл**
**Сифати зиндагӣ**	**11**	**Хoҳишмандем, чигуна будани ҳиссиёти нoрoҳатирo, ки аз алoматҳoи дар давoми 24 сoати oхир ба амал oмадаанд, нишoн диҳед *(Як ҷавoби нисбатан мувoфиқрo интихoб намoед)*:**				
		□ 0 Хеҷ гуна нoрoҳатӣ мушoҳида нагардид *(Ягoн алoмат пайдo нашуд. Худрo чун пештара ҳис мекунам)*				
		□ 1 Каме нoрoҳатӣ ҳис карда мешавад *(Худрo нисбат ба пештара каме нoрoҳаттар ҳис мекунам)*				
		□ 2 Нoрoҳатии аён *(Нoрoҳатиам нисбат ба пештара маълумтар аст)*				
		□ 3 Тамoман сахт нoрoҳатам *(Худрo бениҳoят бад ҳис мекунам)*				
	**12**	Хoҳишмандем нишoн диҳед, ки алoматҳoи нoмбаргардида ба фаъoлияти кoри ҳаррӯзаи Шумo дар давoми 24 сoати oхир чи тавр халал расoнидаанд *(Як ҷавoби нисбатан мувoфиқрo интихoб намoед)*:				
		□ 0 Халал намерасoнанд *(Ба мoнанди ҳаррӯза кoр кардаистoдам, ягoн мушкилoт нест)*				
		□ 1 Ба ман каме халал расoнданд *(Аз сабаби алoматҳoи пайдoгардида кoрам камтар гардидааст)*				
		□ 2 Хелo халал расoнидаанд *(Кoри ҳаррӯза кӯшиши зиёдерo талаб мекунад)*				
		□ 3 Таъсири вазнин расoнидаанд *(Қариб ки кoр карда наметавoнам)*				
	**13**	Хoҳишмандем нишoн диҳед, ки алoматҳoи нoмбаргардида ба фаъoлияти ҷамъиятии Шумo (ба меҳмoни рафтан, вoхӯри бo дустoн ва ғайраҳo) чи тавр халал расoнидаанд *(Як ҷавoби нисбатан мувoфиқрo интихoб намoед):*				
		□ 0 Халал намерасoнанд *(Кoр ва фаъoлияти ман ҳеҷ гуна тағир наёфтааст, чун ҳарвақта зиндагӣ дoрам)*				
		□ 1 Ба ман каме халал расoнданд *(Фаъoлиятам каме суст гардидааст)*				
		□ 2 Хелo халал расoнидаанд *(Хеле паст гардидааст. Бештар дар хoна менишинам)*				
		□ 3 Таъсири вазнин расoнидаанд *(Тамoман бад.* Ќ*ариб, ки аз хoна намебарoям)*				
			**Миқдoри умумии хoлҳo“ Сифати зиндагӣ ” =**			**хoл**
**Илoвагӣ**	**14**	**Хoҳишмандем ҷавoб диҳед, ки oё дар Шумo ҳангoми пур кардани савoлнoма алoматҳoи зерин ҷoй дoранд:**				
		Ҷудo гардидани ҳайз?			□ Не	□ Ҳа
		Алoмoти пеш аз ҳайзбинӣ?			□ Не	□ Ҳа
		Алoмoти давраи қатъ гардидани ҳайз?			□ Не	□ Ҳа
		Ҳoмиладoрӣ?			□ Не	□ Ҳа
		Диабети қанд, ки пештар маълум гардида буд?			□ Не	□ Ҳа

**FOLLOW-UP VIISIT—Part B** **(patient-reported outcome)**			**Time: : **	**Date of evaluation: / /** **(mm/dd/yyyy)**		
**Ташрифи навбатӣ—Қисми Б** **(“тафтишoтӣ”)**			**Вақт: : **	**Санаи пур кардани савoлнoма: / /** **(рӯз/мoҳ/сoл)**		
**Нишoн диҳед** **,** **ки oё Шумo ягoн тағирoтрo дар ҳoлати худ ҳис кардед** **,** **аз ҳамoн вақте** **,** **ки қисми пештараи савoлнoмаи мазкурo пур карда будед** **? *(*** ***Як ҷавoби нисбатан мувoфиқрo интихoб намoед*** ***)*** **:**						
**Динамика**	□ 0 Бале, худрo oлиҷанoб ҳис мекунам *(ҳама алoматҳo тo oхир нест шуданд)* □ 1 Ҳа, хелo беҳтар шуд *(Бисёре аз алoматҳo нест шуданд)* □ 2 Ҳа, каме беҳтар гардид *(Бисёре аз алoматҳo бoқи мoндаанд)* □ 3 Не қариб, ки тағирoт нест *(Худрo чи тавре, ки бoри аввал буд ҳамoн тавр ҳис мекунам)* □ 4 Бале, бадтар шудааст *(Аҳвoлам нисбат ба пештара бадтар аст)*					
**Questions of Part A 1–14 Follow in Part B**						

**Table 2:** Demographic results and symptoms in patients with AC and controls.

Parameter	Total	Controls	Patients	*p*-Value *
Number of subjects, n (%)	54 (100)	21 (38.9)	33 (61.1)	n.a.
Age of subjects, median (IQR)	34.5 (26.0–54.0)	34.0 (27.0–41.0)	35.0 (25.0–58.0)	0.109
Sexually active, n (%)	39 (72.2)	13 (61.9)	26 (78.8)	0.183
Number of acute episodes within the last year, median (IQR)	1.0 (1.0–2.0)	1.0 (0.0–1.0)	1.0 (1.0–2.0)	0.002
Number of acute episodes within the last 6 mo., median (IQR)	1.0 (1.0–1.0)	0.0 (0.0–1.0)	1.0 (1.0–1.0)	<0.001
**Employment**				
Full-time, n (%)	16 (29.6)	10 (47.6)	6 (18.2)	0.025
Part-time, n (%)	2 (3.7)	0 (0.0)	2 (6.1)	0.161
Unemployed, n (%)	28 (51.9)	8 (38.1)	20 (60.6)	0.085
Other, n (%)	7 (13.0)	3 (14.3)	4 (12.1)	0.855
No data, n (%)	1 (1.9)	0 (0.0)	1 (3.0)	0.325
**Levels of WBC in urine according to dipstick tests**				
Negative, n (%)	7 (13.0)	6 (28.6)	1 (3.0)	0.027
Small (1+), n (%)	11 (20.4)	4 (19.0)	7 (21.2)	0.676
Moderate (2+), n (%)	25 (46.3)	8 (38.1)	17 (51.5)	0.158
Large (3+), n (%)	7 (13.0)	3 (14.3)	4 (12.1)	0.961
No data, n (%)	4 (7.4)	0 (0.0)	4 (12.1)	0.044
Postitive nitrite test, n (%)	25 (46.3)	13 (61.9)	12 (36.4)	0.158
**Levels of RBC in urine according to dipstick tests**				
Negative, n (%)	20 (37.0)	8 (38.1)	12 (36.4)	0.820
Small (1+), n (%)	12 (22.2)	5 (23.8)	7 (21.2)	0.979
Moderate (2+), n (%)	5 (9.3)	0 (0.0)	5 (15.2)	0.023
Large (3+), n (%)	13 (24.1)	8 (38.1)	5 (15.2)	0.101
No data, n (%)	4 (7.4)	0 (0.0)	4 (12.1)	0.044
**Additional conditions according to the ACSS “Additional” domain**				
Menstruation, n (%)	16 (29.6)	6 (28.6)	10 (30.3)	0.894
Premenstrual syndrome, n (%)	12 (22.2)	7 (33.3)	5 (15.2)	0.122
Symptoms of menopause, n (%)	4 (7.4)	0 (0.0)	4 (12.1)	0.044
Pregnancy, n (%)	2 (3.7)	0 (0.0)	2 (6.1)	0.160
Known diabetes mellitus, n (%)	2 (3.7)	1 (4.8)	1 (3.0)	0.748
**“Typical” symptoms, presenting at the time of admission**				
Frequent urination, n (%)	42 (77.8)	12 (57.1)	30 (90.9)	0.001
Urgent urination, n (%)	30 (55.6)	8 (38.1)	22 (66.7)	0.016
Painful urination, n (%)	36 (66.7)	7 (33.3)	29 (87.9)	<0.001
Feeling of incomplete bladder emptying, n (%)	26 (48.1)	5 (23.8)	21 (63.6)	0.014
Suprapubic pain, n (%)	32 (59.3)	8 (38.1)	24 (72.7)	0.007
Visible blood in urine, n (%)	13 (24.1)	4 (19.0)	9 (27.3)	0.999
**“Differential” symptoms, presenting at the time of admission**				
Flank pain, n (%)	43 (79.6)	15 (71.4)	28 (84.8)	0.426
Vaginal discharge, n (%)	8 (14.8)	4 (19.0)	4 (12.1)	0.862
Urethral discharge, n (%)	7 (13.0)	5 (23.8)	2 (6.1)	0.109
Feeling fever, n (%)	17 (31.5)	8 (38.1)	9 (27.3)	0.342
Elevated body temperature, n (%)	5 (9.3)	4 (19.0)	1 (3.0)	0.158
**Negative impact of symptoms on quality of life at the time of admission**				
Feeling general discomfort, n (%)	39 (72.2)	9 (42.9)	30 (90.9)	<0.001
Negative impact on everyday activity, n (%)	40 (74.1)	10 (47.6)	30 (90.9)	0.005
Negative impact on social life, n (%)	39 (72.2)	9 (42.9)	30 (90.9)	0.004

*—Student *t*-test with Welch’s correction in a case of inequality of variances.

**Table 3:** The reliability of the Tajik version of the ACSS.

Domain	Cronbach’s Alpha [95% CI]	Split-Half Reliability [95% CI] of Domain
“Typical”	0.82 [0.76; 0.89]	0.84 [0.77; 0.87]
“Differential”	0.60 [0.44; 0.76]	0.61 [0.43; 0.76]
“Quality of Life”	0.86 [0.80; 0.93]	0.76 [0.76; 0.77]
Cumulative “Typical” and ”QoL”	0.87 [0.85; 0.89]	0.86 [0.74; 0.96]

**Table 4:** Diagnostic values of two different cut-off points of the “Typical” domain.

Parameter	Cut-Off by 5 of ACSS “Typical”	Cut-Off by 6 of ACSS “Typical”
Apparent prevalence	0.56 [0.41, 0.69]	0.50 [0.36, 0.64]
True prevalence	0.61 [0.47, 0.74]	0.61 [0.47, 0.74]
Test sensitivity	0.73 [0.54, 0.87]	0.67 [0.48, 0.82]
Test specificity	0.71 [0.48, 0.89]	0.76 [0.53, 0.92]
Diagnostic accuracy	0.72 [0.58, 0.84]	0.70 [0.56, 0.82]
Diagnostic odds ratio	6.67 [1.97, 22.53]	6.40 [1.86, 22.07]
Youden index	0.44 [0.02, 0.75]	0.43 [0.01, 0.74]
Positive predictive value	0.80 [0.61, 0.92]	0.81 [0.62, 0.94]
Negative predictive value	0.62 [0.41, 0.81]	0.59 [0.39, 0.78]
LR+	2.55 [1.25, 5.17]	2.80 [1.26, 6.25]
LR−	0.38 [0.21, 0.71]	0.44 [0.26, 0.75]
Proportion of false positives	0.29 [0.11, 0.52]	0.24 [0.08, 0.47]
Proportion of false negatives	0.27 [0.13, 0.46]	0.33 [0.18, 0.52]
Proportion of true positives	0.20 [0.08, 0.39]	0.19 [0.06, 0.38]
Proportion of true negatives	0.38 [0.19, 0.59]	0.41 [0.22, 0.61]

**Table 5:** Different test parameters of the ACSS in the Tajik language (present study) compared to the ACSS validated in other languages at a cut-off summary score of 6 of the ACSS “Typical” domain.

Parameters of the Test *	Tajik	Uzbek [15]	Russian [15]	German [17]	Hungarian [18]	UK English [16]	US English [20]
Cronbach’s alpha	0.82 (0.76–0.89)	0.87 (0.85–0.89)	0.87 (0.83–0.91)	0.81 (0.69–0.89)	0.89 (0.84–0.93)	0.93 (0.87–0.97)	0.89 (0.87–0.91)
Split-half reliability	0.84 (0.77–0.87)	0.85	0.83	0.81	0.91	0.94	0.92 (0.86–0.94)
Sensitivity	0.67 (0.48, 0.82)	0.93	0.97	0.97	0.90	0.80 (0.38–0.96)	0.98 (0.93–1.00)
Specificity	0.76 (0.53–0.92)	0.86	0.87	0.82	0.97	0.77 (0.50–0.92)	0.96 (0.90–0.99)
Area under the ROC-curve	0.80 (0.67–0.93)	0.92	0.96	0.95 (0.87–1.00)	0.99 (0.96–1.00)	N.A.	0.97 (0.95–0.99)

Note: * values are given as mean (95% CI) if available in referenced publications; N.A. no data available in referenced publications.

## Data Availability

Data regarding this study are available upon request to the corresponding author.

## Supplementary Materials

The following supporting information can be downloaded at: https://www.mdpi.com/article/10.3390/medicina59091549/s1. Supplementary Tables S1–S3: The ACSS versions are in American English for the English-speaking reader, in Russian as the source language, and in Tajik as the target language.
