# Supplementary material for: Linguistic and Clinical Validation of the Tajik Acute Cystitis Symptom Score for Diagnosis and Patient-Reported Outcome in Acute Uncomplicated Cystitis
Source: Medicina (Kaunas). 2023 Aug 25;59(9):1549. doi: 10.3390/medicina59091549 (PMC10534607; doi:10.3390/medicina59091549)
Supplement: Supplementary file 1 [file medicina-59-01549-s001.zip › medicina-2505433-supplementary.pdf]

Linguistic and clinical validation of the Tajik Acute Cystitis Symptom Score questionnaire for diagnostics and patient-reported outcome in acute uncomplicated cystitis

Suppl. Table S1A. Acute Cystitis Symptom Score (ACSS) questionnaire in American English language. Part A.

American English Acute Cystitis Symptom Score (ACSS) – Questionaire

FIRST VISIT – Part A (diagnostic part)

Time:___:__ Date of evaluation: ___/____/_____ (mm/dd/yyyy)

		**Please indicate whether you have had the following symptoms during the past 24 hours, and how severe they were: (Please mark only one answer for each symptom)**	**0**	**1**	**2**	**3**
Typical Symptoms	1	Frequent urination of small amounts of urine *(going to the toilet very often)*	☐ None *up to 4 times per day*	☐ Yes, mild *5-6 times/day*	☐ Yes, moderate *7-8 times/day*	☐ Yes, severe *9-10 or more times/day*
	2	Urgent urination *(a sudden and uncontrollable urge to urinate)*	☐ None	☐ Yes, mild	☐ Yes, moderate	☐ Yes, severe
	3	Feeling burning pain when urinating	☐ None	☐ Yes, mild	☐ Yes, moderate	☐ Yes, severe
	4	Feeling incomplete bladder emptying (*Still feel like you need to urinate after urination*)	☐ None	☐ Yes, mild	☐ Yes, moderate	☐ Yes, severe
	5	Feeling pain not associated with urination in the lower abdomen *(below the belly button)*	☐ None	☐ Yes, mild	☐ Yes, moderate	☐ Yes, severe
	6	Blood seen in urine *(without menses)*	☐ None	☐ Yes, mild	☐ Yes, moderate	☐ Yes, severe
				**Sum of "Typical" scores=**		**points**
Differential	7	Flank pain *(pain in one or both sides of the lower back)*	☐ None	☐ Yes, mild	☐ Yes, moderate	☐ Yes, severe
	8	Abnormal vaginal discharge *(abnormal amount, color and/or odor)*	☐ None	☐ Yes, mild	☐ Yes, moderate	☐ Yes, severe
	9	Discharge from the urethra (*urinary opening*) without urination	☐ None	☐ Yes, mild	☐ Yes, moderate	☐ Yes, severe
	10	Feeling high body temperature/fever<br>Temperature measured ☐ No ☐ Yes	☐ None (≤99.5˚F)	☐ Yes, mild (99.6˚F-100.2˚F)	☐ Yes, moderate (100.3°F-102.0°F)	☐ Yes, severe (≥102.1 ˚F)
				**Sum of "Differential" scores=**		**points**
Quality of Life	11	**Please rate how much discomfort you have experienced because of these symptoms in the past 24 hours *(Please mark only one answer)*:**<br>☐ 0 No discomfort *(No symptoms at all. I feel as good as usual)*<br>☐ 1 Mild discomfort *(I feel a little worse than usual)*<br>☐ 2 Moderate discomfort *(I feel much worse than usual)*<br>☐ 3 Severe discomfort *(I feel terrible)*				
	12	**Please indicate how these symptoms have interfered with your everyday activities/work in the past 24 hours *(Please mark only one answer)*:**<br>☐ 0 Did not interfere at all *(Working as usual on a working day)*<br>☐ 1 Mildly interfered *(Due to the symptoms, I work slightly less)*<br>☐ 2 Moderately interfered *(Daily work requires effort)*<br>☐ 3 Severely interfered *(I almost cannot work)*				
	13	**Please indicate how these symptoms have interfered with your social activities *(visiting people, meeting with friends, etc)* in the past 24 hours *(Please mark only one answer)*:**<br>☐ 0 Did not interfere at all *(My social activities did not change in any way, I live as usual)*<br>☐ 1 Mildly interfered (*Insignificant decrease in activities)*<br>☐ 2 Moderately interfered *(Significant decrease. I have to spend more time at home)*<br>☐ 3 Severely interfered *(It's terrible. I barely left the house)*				
				**Sum of "QoL" scores=**		**points**
Additional	14	**Please indicate whether you have the following at the time of completion of this questionnaire:**				
		Menstruation (Menses)?			☐ No	☐ Yes
		Premenstrual syndrome *(PMS)* ?			☐ No	☐ Yes
		Signs of menopausal syndrome *(e.g. hot flashes)* **?**			☐ No	☐ Yes
		Pregnancy ?			☐ No	☐ Yes
		Known (diagnosed) diabetes mellitus *(high sugar)* ?			☐ No	☐ Yes

Alidjanov et al. Antibiotics (Basel) 2020, 9, 929 [25]

Suppl. Table S1B. Acute Cystitis Symptom Score (ACSS) questionnaire in American English language. Part B.

American English Acute Cystitis Symptom Score (ACSS) – Questionaire

FOLLOW-UP VISIT – Part B (patient-reported outcome)

Time: : Date of evaluation: / / (mm/dd/yyyy)

	Please indicate if you experienced any changes in your symptoms since the first time you completed this questionaire
Dynamics	☐ 0 Yes, I feel back to normal *(All symptoms are completely gone)*<br>☐ 1 Yes, I feel much better *(Most of the symptoms are gone)*<br>☐ 2 Yes, I feel somewhat better *(Only some symptoms are gone)*<br>☐ 3 No, there are barely any changes *(I still have about the same symptoms)*<br>☐ 4 Yes, I feel worse *(My condition is worse)* .

Please indicate whether you have had the following symptoms during the past 24 hours, and how severe they were:

		Please mark only one answer for each symptom	0	1	2	3
Typical Symptoms	1	Frequent urination of small amounts of urine *(going to the toilet very often)*	☐ None<br>*up to 4 times per day*	☐ Yes, mild<br>*5-6 times/day*	☐ Yes, moderate<br>*7-8 times/day*	☐ Yes, severe<br>*9-10 or more*
	2	Urgent urination *(a sudden and uncontrollable urge to urinate)*	☐ None	☐ Yes, mild	☐ Yes, moderate	☐ Yes, severe
	3	Feeling burning pain when urinating	☐ None	☐ Yes, mild	☐ Yes, moderate	☐ Yes, severe
	4	Feeling incomplete bladder emptying (*Still feel like you need to urinate again after urination*)	☐ None	☐ Yes, mild	☐ Yes, moderate	☐ Yes, severe
	5	Feeling pain not associated with urination in the lower abdomen (*below the belly button*)	☐ None	☐ Yes, mild	☐ Yes, moderate	☐ Yes, severe
	6	Blood seen in urine *(without menses)*	☐ None	☐ Yes, mild	☐ Yes, moderate	☐ Yes, severe
				**Sum of "Typical" scores=**		**points**
Differential	7	Flank pain *(pain in one or both sides of the lower back)*	☐ None	☐ Yes, mild	☐ Yes, moderate	☐ Yes, severe
	8	Abnormal vaginal discharge *(abnormal amount, color and/or odor)*	☐ None	☐ Yes, mild	☐ Yes, moderate	☐ Yes, severe
	9	Discharge from the urethra (*urinary opening*) without urination	☐ None	☐ Yes, mild	☐ Yes, moderate	☐ Yes, severe
	10	Feeling high body temperature/fever<br>Temperature measured ☐ No ☐ Yes	☐ None<br>(≤99.5˚F)	☐ Yes, mild<br>(99.6˚F-100.2˚F)	☐ Yes, moderate<br>(100.3˚F-102.0˚F)	☐ Yes, severe<br>(≥102.1 ˚F)
				**Sum of "Differential" scores=**		**points**

Quality of Life	11	**Please rate how much discomfort you have experienced because of these symptoms in the past 24 hours *(Please mark only one answer)*:**<br>☐ 0 No discomfort *(No symptoms at all. I feel as good as usual)*<br>☐ 1 Mild discomfort *(I feel a little worse than usual)*<br>☐ 2 Moderate discomfort *(I feel much worse than usual)*<br>☐ 3 Severe discomfort *(I feel terrible)*
	12	**Please indicate how these symptoms have interfered with your everyday activities/work in the past 24 hours *(Please mark only one answer)*:**<br>☐ 0 Did not interfere at all *(Working as usual on a working day)*<br>☐ 1 Mildly interfered *(Due to the symptoms, I work slightly less)*<br>☐ 2 Moderately interfered *(Daily work requires effort)*<br>☐ 3 Severely interfered *(I almost cannot work)*
	13	**Please indicate how these symptoms have interfered with your social activities *(visiting people, meeting with friends, etc)* in the past 24 hours *(Please mark only one answer)*:**<br>☐ 0 Did not interfere at all *(My social activities did not change in any way, I live as usual)*<br>☐ 1 Mildly interfered *(Insignificant decrease in activities)*<br>☐ 2 Moderately interfered *(Significant decrease. I have to spend more time at home)*<br>☐ 3 Severely interfered *(It's terrible. I barely left the house)*
		**Sum of "QoL" scores= points**

		Please indicate whether you have the following at the time of completion of this questionnaire:		
Additional	14	Menstruation *(Menses)* ?	☐ No	☐ Yes
		Premenstrual syndrome *(PMS)* ?	☐ No	☐ Yes
		Signs of menopausal syndrome *(e.g. hot flashes)* **?**	☐ No	☐ Yes
		Pregnancy ?	☐ No	☐ Yes
		Known *(diagnosed)* diabetes mellitus *(high sugar)* ?	☐ No	☐ Yes

Alidjanov et al. Antibiotics (Basel) 2020, 9, 929 [21]

Linguistic and clinical validation of the Tajik Acute Cystitis Symptom Score
Supplementary Table S2

Suppl. Table S2A. Acute Cystitis Symptom Score (ACSS) questionnaire in Russian language. Part A.

Анкета ACSS

Первое посещение - Часть А ("диагностическая")

Время: ____чч:____мм Дата обследования:__/__/ (дд/мм/гггг)

		Пожалуйста, укажите, отмечали ли Вы следующие симптомы в течение последних 24 часов, и оцените степень их Выраженности (Только один ответ для каждого пункта):	0	1	2	3
Типичные симптомы	1	Учащенное мочеиспускание малыми объемами мочи (частое посещение туалета)	☐ Нет *до 4 раз в день*	☐ Да, слабо 5-6 раз в день	☐ Да, умеренно 7-8 раз в день	☐ Да, сильно 9-10 раз в день и чаще
	2	Срочные (сильные и неудержимые) позывы к мочеиспусканию	☐ Нет	☐ Да, слабо	☐ Да, умеренно	☐ Да, сильно
	3	Боль или жжение при мочеиспускании	☐ Нет	☐ Да, слабо	☐ Да, умеренно	☐ Да, сильно
	4	Чувство неполного опорожнения мочевого пузыря	☐ Нет	☐ Да, слабо	☐ Да, умеренн	☐ Да, сильно
	5	*Боль или дискомфорт внизу живота (надлобковой области)*	☐ Нет	☐ Да, слабо	☐ Да, умеренно	☐ Да, сильно
	6	Наличие крови в моче	☐ Нет	☐ Да, слабо	☐ Да, умеренно	☐ Да, сильно
		**Суммарный балл "Типичных":**				**баллов**
Дифференциальные	7	Боль в поясничной области (может быть односторонней)	☐ Нет	☐ Да, слабо	☐ Да, умеренно	☐ Да, сильно
	8	Гнойные выделения из половых путей (особенно по утрам)	☐ Нет	☐ Да, слабо	☐ Да, умеренно	☐ Да, сильно
	9	Гнойные выделения из мочевыводящих путей (вне акта мочеиспускания)	☐ Нет	☐ Да, слабо	☐ Да, умеренно	☐ Да, сильно
	10	Озноб (познабливание) /чувство повышенной температуры тела *(Ha mérte, kérem jelölje be az értéket)* (Если измеряли, укажите значения)	☐ Нет ≤37.5 ˚C	☐ Да, слабо 37.6-37.9 ˚C	☐ Да, умеренно 38.0-38.9 ˚C	☐ Да, сильно ≥39.0 ˚C
		**Суммарный балл “Дифференциальных”:**				**баллов**

Качество жизни

11 **Пожалуйста, укажите, насколько было выражено чувство дискомфорта, вызванное вышеуказанными симптомами, в течение последних 24 часов (Отметьте один, наиболее подходящий ответ):**

- ☐ 0 Никакого дискомфорта (Нет никаких симптомов. Чувствую себя как обычно)
- ☐ 1 Чуть заметный дискомфорт (Чувствую себя чуть хуже обычного)
- ☐ 2 Выраженный дискомфорт (Чувствую себя заметно хуже обычного)
- ☐ 3 Очень сильный дискомфорт (Чувствую себя ужасно)

12 **Пожалуйста, укажите, насколько вышеуказанные симптомы мешали Вашей повседневной активности/ работоспособности в течение последних 24 часов (Отметьте один, наиболее подходящий ответ):**

- ☐ 0 Нисколько не мешали (Работаю как в обычные дни, без затруднений)
- ☐ 1 Мешали незначительно (Из-за возникших симптомов, работаю чуть меньше)
- ☐ 2 Значительно мешали (Повседневная работа требует больших усилий)
- ☐ 3 Ужасно мешали (Практически не могу работать)

13 **Пожалуйста, укажите, насколько вышеуказанные симптомы мешали Вашей общественной активности (поход в гости, встречи с друзьями и т.п.) в течение последних 24 часов (Отметьте один, наиболее подходящий ответ):**

- ☐ 0 Нисколько не мешали (Моя деятельность и активность никоим образом не изменились, живу как обычно)
- ☐ 1 Мешали незначительно (Незначительное снижение деятельности)
- ☐ 2 Значительно мешали (Значительное снижение. Больше сижу дома)
- ☐ 3 Ужасно мешали (Ужасно. Практически не выходила из дому)

Суммарный балл "Качества жизни": баллов

Дополнительные

14 **Пожалуйста, ответьте, имеются ли у Вас на момент заполнения анкеты следующие:**

Менструальные выделения?	☐ Нет	☐ Да
Так называемый «предменструальный синдром» (ПМС) ?	☐ Нет	☐ Да
Признаки климактерического синдрома?	☐ Нет	☐ Да
Беременность?	☐ Нет	☐ Да
Сахарный диабет, выявленный ранее?	☐ Нет	☐ Да

Alidjanov et al. Urologiia 2014, 6, 14–22 [15]

Suppl. Table S2B. Acute Cystitis Symptom Score (ACSS) questionnaire in Russian language. Part B.

Анкета ACSS

Контрольное посещение - Часть Б ("диспансерная")

Время: ____чч:____мм Дата обследования:__/__/__ (дд/мм/гггг)

Укажите, отметили ли Вы какие-либо изменения в своем состоянии с тех пор, как Вы заполнили предыдущую часть данной анкеты? (Отметьте один, наиболее подходящий ответ) :

Динамика	
	☐ 0 Да, чувствую себя отлично (Все симптомы прошли окончательно)
	☐ 1 Да, стало заметно лучше (Большинство симптомов исчезло)
	☐ 2 Да, стало несколько лучше (Большинство симптомов всё еще присутствует)
	☐ 3 Нет, изменений практически нет (Чувствую себя так же, как и в прошлый раз)
	☐ 4 Да, стало хуже (Мое состояние хуже, чем в прошлый раз)

Пожалуйста, укажите, отмечали ли Вы следующие симптомы в течение последних 24 часов, и оцените степень их Выраженности (Только один ответ для каждого пункта)

			0	1	2	3
Типичные симптомы	1	Учащенное мочеиспускание малыми объемами мочи (частое посещение туалета)	☐ Нет *до 4 раз в день*	☐ Да, слабо 5-6 раз в день	☐ Да, умеренно 7-8 раз в день	☐ Да, сильно 9-10 раз в день и чаще
	2	Срочные (сильные и неудержимые) позывы к мочеиспусканию	☐ Нет	☐ Да, слабо	☐ Да, умеренно	☐ Да, сильно
	3	Боль или жжение при мочеиспускании	☐ Нет	☐ Да, слабо	☐ Да, умеренно	☐ Да, сильно
	4	Чувство неполного опорожнения мочевого пузыря	☐ Нет	☐ Да, слабо	☐ Да, умеренно	☐ Да, сильно
	5	*Боль или дискомфорт внизу живота (надлобковой области)*	☐ Нет	☐ Да, слабо	☐ Да, умеренно	☐ Да, сильно
	6	Наличие крови в моче	☐ Нет	☐ Да, слабо	☐ Да, умеренно	☐ Да, сильно
					**Суммарный балл "Типичных":**	**баллов**
Дифференциальные	7	Боль в поясничной области (может быть односторонней)	☐ Нет	☐ Да, слабо	☐ Да, умеренно	☐ Да, сильно
	8	Гнойные выделения из половых путей (особенно по утрам)	☐ Нет	☐ Да, слабо	☐ Да, умеренно	☐ Да, сильно
	9	Гнойные выделения из мочевыводящих путей (вне акта мочеиспускания)	☐ Нет	☐ Да, слабо	☐ Да, умеренно	☐ Да, сильно
	10	Озноб (познабливание) /чувство повышенной температуры тела *(Ha mérte, kérem jelölje be az értéket)* (Если измеряли, укажите значения)	☐ Нет ≤37.5 ˚C	☐ Да, слабо 37.6-37.9 ˚C	☐ Да, умеренно 38.0-38.9 ˚C	☐ Да, сильно ≥39.0 ˚C
					**Суммарный балл “Дифференциальных”:**	**баллов**

Качество жизни

11 Пожалуйста, укажите, насколько было выражено чувство дискомфорта, вызванное вышеуказанными симптомами, в течение последних 24 часов (Отметьте один, наиболее подходящий ответ):

☐ 0 Никакого дискомфорта (Нет никаких симптомов. Чувствую себя как обычно)
☐ 1 Чуть заметный дискомфорт (Чувствую себя чуть хуже обычного)
☐ 2 Выраженный дискомфорт (Чувствую себя заметно хуже обычного)
☐ 3 Очень сильный дискомфорт (Чувствую себя ужасно)

12 Пожалуйста, укажите, насколько вышеуказанные симптомы мешали Вашей повседневной активности/ работоспособности в течение последних 24 часов (Отметьте <u>один</u>, наиболее подходящий ответ):

☐ 0 Нисколько не мешали (Работаю как в обычные дни, без затруднений)
☐ 1 Мешали незначительно (Из-за возникших симптомов, работаю чуть меньше)
☐ 2 Значительно мешали (Повседневная работа требует больших усилий)
☐ 3 Ужасно мешали (Практически не могу работать)

13 Пожалуйста, укажите, насколько вышеуказанные симптомы мешали Вашей общественной активности (поход в гости, встречи с друзьями и т.п.) в течение последних 24 часов (Отметьте <u>один</u>, наиболее подходящий ответ):

☐ 0 Нисколько не мешали (Моя деятельность и активность никоим образом не изменились, живу как обычно)
☐ 1 Мешали незначительно (Незначительное снижение деятельности)
☐ 2 Значительно мешали (Значительное снижение. Больше сижу дома)
☐ 3 Ужасно мешали (Ужасно. Практически не выходила из дому)

Суммарный балл "Качества жизни": баллов

Дополнительн

14 Пожалуйста, ответьте, имеются ли у Вас на момент заполнения анкеты следующие:

Менструальные выделения?	☐ Нет	☐ Да
Так называемый «предменструальный синдром» (ПМС) ?	☐ Нет	☐ Да
Признаки климактерического синдрома?	☐ Нет	☐ Да
Беременность?	☐ Нет	☐ Да
Сахарный диабет, выявленный ранее?	☐ Нет	☐ Да

Alidjanov et al. Urologiia 2014, 6, 14–22 [15]

Suppl. Table S3A. Tajik Version of the Acute Cystitis Symptom Score (ACSS) – Part A

Саволномаи ACSS

Ташрифи аввалин - Қисми А ("ташхисӣ")

Вақт: _____:_____ Санаи пур кардани саволнома: / / (рӯз/моҳ/сол)

		**Хоҳишмандем нишон диҳед, ки оё Шумо аломатҳои зеринро дар давоми 24 соати охир ҳис намудед ва дараҷаи таъсири онҳоро баҳо диҳед *(Танҳо барои ҳар як аломат як ҷавоб диҳед)*:**				
			**0**	**1**	**2**	**3**
**Аломатҳои типӣ**	1	Тез-тез пешобкунӣ бо ҳаҷми кам *(тез-тез ба ҳоҷатхона рафтан)*	☐ Не *то 4 маротиба дар 1 рӯз*	☐ Бале, нисбат ба ҳарвақта бештар *5-6 маротиба дар 1 рӯз*	☐ Бале, зудтар пайдо мешавад *7-8 маротиба дар 1 рӯз*	☐ Бале, тез-тез пайдо мешавад *9-10 маротиба дар 1 рӯз*
	2	Майли бетоқат пешобкунӣ (*сахт ва нигоҳдоштанашаванда)*	☐ Не	☐ Ҳа, суст	☐ Ҳа, муътадил	☐ Ҳа, сахт
	3	Дард ё сӯзиш ҳангоми пешобкунӣ	☐ Не	☐ Ҳа, суст	☐ Ҳа, муътадил	☐ Ҳа, сахт
	4	Ҳиссиёти пурра холи нашудани пешобдон	☐ Не	☐ Ҳа, суст	☐ Ҳа, муътадил	☐ Ҳа, сахт
	5	Дард ва нороҳати дар қисми поёни шикам *(дар қисми болоии зери ноф)*	☐ Не	☐ Ҳа, суст	☐ Ҳа, муътадил	☐ Ҳа, сахт
	6	Мавҷуд будани хун дар пешоб	☐ Не	☐ Ҳа, суст	☐ Ҳа, муътадил	☐ Ҳа, сахт
				**Миқдори умумии холҳо " Типӣ "=**		**хол**
**Дифференциалӣ**	7	Дард дар қисмати камарбанд*	☐ Не	☐ Ҳа, суст	☐ Ҳа, муътадил	☐ Ҳа, сахт
	8	Пайдошавии зардоб дар узвҳои таносул *(асосан саҳаргоҳон)*	☐ Не	☐ Ҳа, суст	☐ Ҳа, муътадил	☐ Ҳа, сахт
	9	Пайдошавии зардоб аз роҳҳои пешобрав *(новобаста аз пешобкунӣ)*	☐ Не	☐ Ҳа, суст	☐ Ҳа, муътадил	☐ Ҳа, сахт
	10	Ҳарорати баланди бадан *(бештар аз 37,5° С)*/ларза *(Агар чен карда бошед интихоб намоед)*	☐ Не ≤37.5 ˚C	☐ Ҳа, суст 37.6-37.9 ˚C	☐ Ҳа, муътадил 38.0-38.9 ˚C	☐ Ҳа, сахт ≥39.0 ˚C
		* - бисёртар аз як тараф		**Миқдори умумии холҳо " Дифференциалӣ "=**		**хол**

**Сифати зиндагӣ**	11	**Хоҳишмандем, чигуна будани ҳиссиёти нороҳатиро, ки аз аломатҳои дар давоми 24 соати охир ба амал омадаанд, нишон диҳед (*Як ҷавоби нисбатан мувофиқро интихоб намоед)*:**
		☐ 0 Хеҷ гуна нороҳатӣ мушоҳида нагардид *(Ягон аломат пайдо нашуд. Худро чун пештара ҳис мекунам)*
		☐ 1 Каме нороҳатӣ ҳис карда мешавад *(Худро нисбат ба пештара каме нороҳаттар ҳис мекунам)*
		☐ 2 Нороҳатии аён *(Нороҳатиам нисбат ба пештара маълумтар аст)*
		☐ 3 Тамоман сахт нороҳатам *(Худро бениҳоят бад ҳис мекунам)*
	12	**Хоҳишмандем нишон диҳед, ки аломатҳои номбаргардида ба фаъолияти кори ҳаррӯзаи Шумо дар давоми 24 соати охир чи тавр халал расонидаанд (*Як ҷавоби нисбатан мувофиқро интихоб намоед)*:**
		☐ 0 Халал намерасонанд *(Ба монанди ҳаррӯза кор кардаистодам, ягон мушкилот нест)*
		☐ 1 Ба ман каме халал расонданд *(Аз сабаби аломатҳои пайдогардида корам камтар гардидааст)*
		☐ 2 Хело халал расонидаанд *(Кори ҳаррӯза кӯшиши зиёдеро талаб мекунад)*
		☐ 3 Таъсири вазнин расонидаанд *(Қариб ки кор карда наметавонам)*
	13	**Хоҳишмандем нишон диҳед, ки аломатҳои номбаргардида ба фаъолияти ҷамъиятии Шумо (ба меҳмони рафтан, вохӯри бо дустон ва ғайраҳо) чи тавр халал расонидаанд (*Як ҷавоби нисбатан мувофиқро интихоб намоед):***
		☐ 0 Халал намерасонанд *(Кор ва фаъолияти ман ҳеҷ гуна тағир наёфтааст, чун ҳарвақта зиндагӣ дорам)*
		☐ 1 Ба ман каме халал расонданд *(Фаъолиятам каме суст гардидааст)*
		☐ 2 Хело халал расонидаанд *(Хеле паст гардидааст. Бештар дар хона менишинам)*
		☐ 3 Таъсири вазнин расонидаанд *(Тамоман бад. Ќариб, ки аз хона намебароям)*
		**Миқдори умумии холҳо" Сифати зиндагӣ " = хол**

**Иловагӣ**	14	**Хоҳишмандем ҷавоб диҳед, ки оё дар Шумо ҳангоми пур кардани саволнома аломатҳои зерин ҷой доранд:**		
		Ҷудо гардидани ҳайз?	☐ Не	☐ Ҳа
		Аломоти пеш аз ҳайзбинӣ?	☐ Не	☐ Ҳа
		Аломоти давраи қатъ гардидани ҳайз?	☐ Не	☐ Ҳа
		Ҳомиладорӣ?	☐ Не	☐ Ҳа
		Диабети қанд, ки пештар маълум гардида буд?	☐ Не	☐ Ҳа

Suppl. Table S3B. Tajik Version of the Acute Cystitis Symptom Score (ACSS) – Part B

Саволномаи ACSS

Ташрифи навбатӣ - Қисми Б ("тафтишотӣ")

Вақт: _____:_____ Санаи пур кардани саволнома: / / (рӯз/моҳ/сол)

**Нишон диҳед, ки оё Шумо ягон тағиротро дар ҳолати худ ҳис кардед, аз ҳамон вақте, ки қисми пештараи саволномаи мазкуро пур карда будед? (*Як ҷавоби нисбатан мувофиқро интихоб намоед*):**	
**Динамика**	☐ 0 Бале, худро оличаноб ҳис мекунам (*ҳама аломатҳо то охир нест шуданд*)<br>☐ 1 Ҳа, хело беҳтар шуд (*Бисёре аз аломатҳо нест шуданд*)<br>☐ 2 Ҳа, каме беҳтар гардид (*Бисёре аз аломатҳо боқи мондаанд*)<br>☐ 3 Не қариб, ки тағирот нест (*Худро чи тавре, ки бори аввал буд ҳамон тавр ҳис мекунам*)<br>☐ 4 Бале, бадтар шудааст (*Аҳволам нисбат ба пештара бадтар аст*)

Хоҳишмандем нишон диҳед, ки оё Шумо аломатҳои зеринро дар давоми 24 соати охир ҳис намудед ва дараҷаи таъсири онҳоро баҳо диҳед (*Танҳо барои ҳар як аломат як ҷавоб диҳед*):

	№		0	1	2	3
**Аломатҳои типӣ**	1	Тез-тез пешобкунӣ бо ҳаҷми кам (*тез-тез ба ҳоҷатхона рафтан*)	☐ Не<br>*то 4 маротиба дар 1 рӯз*	☐ Бале, нисбат ба ҳарвақта бештар<br>*5-6 маротиба дар 1 рӯз*	☐ Бале, зудтар пайдо мешавад<br>*7-8 маротиба дар 1 рӯз*	☐ Бале, тез-тез пайдо мешавад<br>*9-10 маротиба дар 1 рӯз*
	2	Майли бетоқат пешобкунӣ (*сахт ва нигоҳдоштанашаванда*)	☐ Не	☐ Ҳа, суст	☐ Ҳа, муътадил	☐ Ҳа, сахт
	3	Дард ё сӯзиш ҳангоми пешобкунӣ	☐ Не	☐ Ҳа, суст	☐ Ҳа, муътадил	☐ Ҳа, сахт
	4	Ҳиссиёти пурра холи нашудани пешобдон	☐ Не	☐ Ҳа, суст	☐ Ҳа, муътадил	☐ Ҳа, сахт
	5	Дард ва нороҳати дар қисми поёни шикам (*дар қисми болоии зери ноф*)	☐ Не	☐ Ҳа, суст	☐ Ҳа, муътадил	☐ Ҳа, сахт
	6	Мавҷуд будани хун дар пешоб	☐ Не	☐ Ҳа, суст	☐ Ҳа, муътадил	☐ Ҳа, сахт
				**Миқдори умумии холҳо " Типӣ "=**		**хол**
**Дифференциалӣ**	7	Дард дар қисмати камарбанд*	☐ Не	☐ Ҳа, суст	☐ Ҳа, муътадил	☐ Ҳа, сахт
	8	Пайдошавии зардоб дар узвҳои таносул (*асосан саҳаргоҳон*)	☐ Не	☐ Ҳа, суст	☐ Ҳа, муътадил	☐ Ҳа, сахт
	9	Пайдошавии зардоб аз роххои пешобрав (*новобаста аз пешобкунӣ*)	☐ Не	☐ Ҳа, суст	☐ Ҳа, муътадил	☐ Ҳа, сахт
	10	Ҳарорати баланди бадан (*бештар аз 37,5 °C*)/ларза<br>*(Агар чен карда бошед интихоб намоед)*	☐ Не<br>≤37.5˚ C	☐ Ҳа, суст<br>37.6-37.9˚ C	☐ Ҳа, муътадил<br>38.0-38.9 ˚ C	☐ Ҳа, сахт<br>≥39.0˚ C
		* - бисёртар аз як тараф		**Миқдори умумии холҳо " Дифференциалӣ "=**		**хол**

	№	
**Сифати зиндагӣ**	11	**Хоҳишмандем чигуна будани ҳиссиёти нороҳатиро, ки аз аломатҳои дар давоми 24 соати охир ба амал омадаанд, нишон диҳед (*Як ҷавоби нисбатан мувофиқро интихоб намоед*):**
		☐ 0 Хеҷ гуна нороҳатӣ мушоҳида нагардид (*Ягон аломат пайдо нашуд. Худро чун пештара ҳис мекунам*)<br>☐ 1 Каме нороҳатӣ ҳис карда мешавад (*Худро нисбат ба пештара каме нороҳаттар ҳис мекунам*)<br>☐ 2 Нороҳатии аён (*Нороҳатиам нисбат ба пештара маълумтар аст*)<br>☐ 3 Тамоман сахт нороҳатам (*Худро бениҳоят бад ҳис мекунам*)
	12	**Хоҳишмандем нишон диҳед, ки аломатҳои номбаргардида ба фаъолият кори ҳаррӯзаи Шумо дар давоми 24 соати охир чи тавр халал расонидаанд (*Як ҷавоби нисбатан мувофиқро интихоб намоед*):**
		☐ 0 Халал намерасонанд (*Ба монанди ҳаррӯза кор карда истодам, ягон мушкилот нест*)<br>☐ 1 Ба ман каме халал расонданд (*Аз сабаби аломатҳои пайдогардида корам камтар гардидааст*)<br>☐ 2 Хело халал расонидаанд (*Кори ҳаррӯза кӯшиши зиёдеро талаб мекунад*)<br>☐ 3 Таъсири вазнин расонидаанд (*Қариб ки кор карда наметавонам*)
	13	**Хоҳишмандем нишон диҳед, ки аломатҳои номбаргардида ба фаъолияти ҷамъиятии Шумо (ба меҳмони рафтан, вохӯри бо дустон ва ғайраҳо) чи тавр халал расонидаанд (*Як ҷавоби нисбатан мувофиқро интихоб намоед*):**
		☐ 0 Халал намерасонанд (*Кор ва фаъолияти ман ҳеҷ гуна тағир наёфтааст, чун ҳарвақта зиндаги дорам*)<br>☐ 1 Ба ман каме халал расонданд (*Фаъолиятам каме суст гардидааст*)<br>☐ 2 Хело халал расонидаанд (*Хеле паст гардидааст. Бештар дар хона мешинам*)<br>☐ 3 Таъсири вазнин расонидаанд (*Тамоман бад. Қариб, ки аз хона намебароям*)
		**Миқдори умумии холҳо" Сифати зиндагӣ " = хол**

	№			
**Иловагӣ**	14	**Хоҳишмандем ҷавоб диҳед, ки оё дар Шумо ҳангоми пур кардани саволнома аломатҳои зерин ҷой доранд:**		
		Ҷудо гардидани ҳайз?	☐ Не	☐ Ҳа
		Аломоти пеш аз ҳайзбинӣ?	☐ Не	☐ Ҳа
		Аломоти давраи қатъ гардидани ҳайз?	☐ Не	☐ Ҳа
		Ҳомиладорӣ?	☐ Не	☐ Ҳа
		Диабети қанд, ки пештар маълум гардида буд?	☐ Не	☐ Ҳа
